# Screening for distant metastases in patients with ipsilateral breast tumor recurrence: the impact of different imaging modalities on distant recurrence-free interval

**DOI:** 10.1007/s10549-019-05205-z

**Published:** 2019-04-06

**Authors:** Ingrid G. M. Poodt, Robert-Jan Schipper, Bianca T. A. de Greef, Guusje Vugts, Adriana J. G. Maaskant-Braat, Frits H. Jansen, Dirk N. J. Wyndaele, Adri C. Voogd, Grard A. P. Nieuwenhuijzen, the Sentinel Node And Recurrent Breast Cancer (SNARB) Research Group, R. M. H. Roumen, E. J. T. Luiten, E. J. T. Rutgers, M. T. F. D. Vrancken-Peeters, M. Bessems, J. M. Klaase, S. Muller, A. B. Francken, T. Van Dalen, L. Jansen, S. A. Koopal, Y. L. J. Vissers, M. L. Smidt, J. W. S. Merkus, C. M. E. Contant, P. H. Veldman, E. M. H. Linthorst-Niers, J. R. van der Sijp, O. R. Guicherit, L. B. Koppert, A. M. Bosch, L. J. A. Strobbe, M. S. Schlooz-Vries, I. E. Arntz, J. A. van Essen, J. W. D. de Waard, B. C. Vrouenraets, B. van Ooijen

**Affiliations:** 1 Department of Surgery, Catharina Hospital, Michelangelolaan 2, 5623 EJ Eindhoven, The Netherlands; 2 Department of Clinical Epidemiology and Medical Technology Assessment, Maastricht University Medical Center, Maastricht, The Netherlands; 3 Department of Surgery, Máxima Medical Center, Veldhoven, Eindhoven, The Netherlands; 4 Department of Radiology, Catharina Hospital, Eindhoven, The Netherlands; 5 Department of Nuclear Medicine, Catharina Hospital, Eindhoven, The Netherlands; 6 Department of Epidemiology, Faculty of Health Medicine and Life Sciences, Research Institute Growth and Development (GROW), Maastricht University, Maastricht, The Netherlands; 7 Utrecht Cancer Registry, Netherlands Comprehensive Cancer Organization, Utrecht, The Netherlands

**Keywords:** Breast cancer, Ipsilateral breast tumor recurrence, Preoperative screening, Conventional imaging, ^18^F-FDG PET-CT, Distant metastasis, Propensity score weight

## Abstract

**Purpose:**

In patients with ipsilateral breast tumor recurrence (IBTR), the detection of distant disease determines whether the intention of the treatment is curative or palliative. Therefore, adequate preoperative staging is imperative for optimal treatment planning. The aim of this study is to evaluate the impact of conventional imaging techniques, including chest X-ray and/or CT thorax-(abdomen), liver ultrasonography(US), and skeletal scintigraphy, on the distant recurrence-free interval (DRFI) in patients with IBTR, and to compare conventional imaging with ^18^F-FDG PET-CT or no imaging at all.

**Methods:**

This study was exclusively based on the information available at time of diagnoses of IBTR. To adjust for differences in baseline characteristics between the three imaging groups, a propensity score (PS) weighted method was used.

**Results:**

Of the 495 patients included in the study, 229 (46.3%) were staged with conventional imaging, 89 patients (19.8%) were staged with ^18^F-FDG PET-CT, and in 168 of the patients (33.9%) no imaging was used (*N* = 168). After a follow-up of approximately 5 years, 14.5% of all patients developed a distant recurrence as first event after IBTR. After adjusting for the PS weights, the Cox regression analyses showed that the different staging methods had no significant impact on the DRFI.

**Conclusions:**

This study showed a wide variation in the use of imaging modalities for staging IBTR patients in the Netherlands. After using PS weighting, no statistically significant impact of the different imaging modalities on DRFI was shown. Based on these results, it is not possible to recommend staging for distant metastases using ^18^F-FDG PET-CT over conventional imaging techniques.

**Electronic supplementary material:**

The online version of this article (10.1007/s10549-019-05205-z) contains supplementary material, which is available to authorized users.

## Introduction

In patients with ipsilateral breast tumor recurrence (IBTR), adequate preoperative staging is imperative for tailoring optimal treatment plans. The absence or presence, and if so, the extensiveness of distant metastases determines the curative or palliative intent of the treatment of patients with IBTR [1]. To evaluate for distant metastases at the time of IBTR, preoperative staging is recommended [2–4]. Conventional imaging, including chest X-ray and/or CT thorax-(abdomen), ultrasonography (US) of the liver, and skeletal scintigraphy, is the standard of care in many hospitals [2–4].

Besides these conventional imaging techniques, studies have focused on the value of 18-Flurorine-2-Fluoro-2-deoxy-D-Glucose positron emission tomography (^18^F-FDG PET) and ^18^F-FDG PET computer tomography (^18^F-FDG PET-CT) in patients with cancer [5–8]. The combined ^18^F-FDG PET and CT technique provides anatomical and functional information, and has been demonstrated to be an accurate technique in staging patients with IBTR and for the detection of distant metastases [4, 9–12]. In a systematic review, Pennant et al. reported a sensitivity of 96% and specificity of 89% for ^18^F-FDG PET-CT [7]. Furthermore, ^18^F-FDG PET-CT is able to screen the whole body, including regional lymph nodes, in one session and may reduce the need for additional diagnostic procedures often needed to further analyze lesions found on conventional staging images [13].

However, ^18^F-FDG PET-CT is an expensive procedure and false-positive outcomes, caused for example by inflammatory processes, physiological muscle uptake, or old fracture sites, could result in unnecessary additional procedures as well [7]. False-negative results can occur due to low FDG uptake in some conditions, such as invasive lobular carcinoma, ongoing endocrine therapy, and small highly sclerotic skeletal lesions with low rate of actively replicating cells [14]. The clinical role of ^18^F-FDG PET-CT remains controversial [15], since there is no evidence of the impact of ^18^F-FDG PET-CT on patients’ outcomes. Guidelines are still quite conservative in recommending the use of ^18^F-FDG PET-CT as first tool for screening distant metastases, in both the primary and recurrent setting.

Theoretically, one could expect that patients screened with a more sensitive screening technique, such as ^18^F-FDG PET-CT [7], would experience less distant recurrences during follow-up or would experience these later during follow-up compared to patients screened with conventional imaging or no staging at all. Besides, with a more sensitive screening technique less patients would receive unnecessary curative treatments associated with high morbidity. Therefore, the aim of this study is to evaluate the impact of conventional imaging versus ^18^F-FDG PET-CT versus no imaging, on distant recurrence-free interval in patients diagnosed with IBTR, in order to optimize treatment planning.

## Patients and methods

### SNARB-study design

The Sentinel Node and Recurrent Breast cancer (SNARB) study is a multicenter national registration study in which 36 Dutch hospitals participated [16, 17]. Patients with clinically apparent ipsilateral or contralateral lymph node metastases and patients with distant metastases at the time of diagnosis of IBTR were excluded. A total of 536 patients with IBTR were included in the SNARB study. No obligatory requirements were formulated in the protocol regarding the use of imaging modalities to stage patients diagnosed with an IBTR.

### Patients

All patients with IBTR, treated with a curative intent, and staged cTxN0M0 were considered eligible for inclusion. All included patients were divided into three groups according to the preoperative staging procedure: conventional imaging, ^18^F-FDG PET-CT, or no staging at all. Conventional imaging included the use of the following imaging techniques: (1) chest X-ray and/or CT thorax-(abdomen), (2) ultrasonography (US) of the liver or CT thorax-(abdomen), and (3) skeletal scintigraphy. Patients in the conventional imaging group who did not undergo a scan of the thorax, liver, and skeletal scintigraphy were excluded.

### Definition of recurrences

Distant recurrences (DR) were defined as any evidence of disease outside the ipsilateral breast, contralateral breast, and regional lymph nodes. A regional recurrence (RR) was defined as any evidence of disease found in ipsilateral intramammary nodes, ipsi- and contralateral internal mammary nodes, ipsi- and contralateral axillary nodes, and ipsi- and contralateral infra- and supra-clavicular nodes [18, 19]. Lymph node recurrences found outside these nodal basins were defined as distant metastatic disease. An event in the contralateral breast was defined as a new primary tumor and was not considered a recurrence, unless it could be proven that it was metastatic disease [20].

### Follow-up

In 2017, follow-up of the 536 patients in the SNARB study was updated. General practitioners were actively contacted for additional follow-up information when hospital records showed no outpatient clinic visits for more than 1 year. Date of last follow-up was documented as last visit to the outpatient clinic, date of last visit to the general practitioner, or date of death in case the patient had deceased. Follow-up time was defined as the time between date of surgery for IBTR and date of last follow-up.

Distant recurrence-free interval (DRFI) was defined as the time between date of IBTR surgery and date of diagnosis of a DR or date of last follow-up. Only distant recurrences developing as first event after IBTR were recorded as a distant event. Distant recurrences occurring after a local or regional re-recurrence following IBTR were censored.

### Statistics

Only the information available at time of diagnosis of IBTR, so before systemic staging imaging, was used for the analyses. The baseline characteristics were compared between the three staging groups. Categorical variables were tested with a Chi-square statistics or Fisher exact test when necessary and continuous variables were tested with a one-way ANOVA analysis. A 2-sided *p* value of < 0.05 was considered statistically significant.

The effect of the method of staging on the DRFI was analyzed with the use of propensity score (PS) adjustment. A PS was calculated for every patient, based on the possible confounders. Because the outcome of interest was divided in three staging methods groups, multinomial propensity scores were used. The propensity scores were calculated with the MNPS package, an extended version of the TWANG package, in R. To obtain the propensity score weights for multiple staging methods, a generalized boosted model (GBM) regression was used. The GBM was used with 3000 number of trees and different stopping rules were checked: the mean effect size, maximal effect size, mean Kolmogorov–Smirnov, and maximal Kolmogorov–Smirnov. For the specific research question, the average treatment estimation (ATE) comparison was used.

By using the GBM model, the overlaps between the three groups were checked. In practice, overlapping meant that every patient could have received each staging modality and that no values of the covariates occurred only in one of the staging groups. Box plots were used for comparing the distribution of propensity scores and testing the overlap. Generally, standardized mean differences of less than 0.20 were considered small (which is good), 0.40 were considered moderate, and 0.60 were considered large.

Next to the overlap, also the balance of the three groups was assessed. Finally, a combination of the overlap plot, the balance plots, and covariate table were used to assess whether the groups were sufficiently similar to support causal estimation of the primary research question.

After the propensity score weights were calculated, the weights were used in a weighted survival analysis to calculate the effect of the staging method on the DRFI. The effect was investigated with the use of Kaplan–Meier curves and tested with a Cox regression model. When a covariate was still unbalanced after PS weighting, the covariate was included in the Cox regression model to correct for it [21–23].

## Results

### Patients

Of the 536 patients for whom follow-up data were collected, 21 (4%) were lost to follow-up due to emigration, lack of information, or withdrawal of informed consent. Of the 515 (96%) remaining patients, 20 patients were only partly screened for distant metastases and therefore excluded, resulting in a study cohort of 495 patients (92.4%).

The median age at the time of IBTR was 64.0 years (range 26–93). The median time from primary surgery to diagnosis of IBTR (DFI) was 10.6 years (range 0.4–32). The majority of the patients had a primary tumor ≤ 2 cm (55.4%), a primary negative nodal status (72.1%, as determined by sentinel lymph node biopsy and/or axillary lymph node dissection), and hormone receptor-positive, human epidermal growth receptor 2 (HER2)-negative (67.9%). The different covariates were divided per staging group are shown in Table 1, with the corresponding *p* values (unadjusted *p* value). In Table 1, it is shown that all variables are statistically significant, except for the median time from primary surgery to IBTR diagnose. In the ^18^F-FDG PET-CT group, more patients were primary treated with a mastectomy, and more patients had primary positive lymph nodes. In the no-staged group, the patients had a higher mean age at time of IBTR (66 years vs. 63 years in the conventional group and 62.5 years in the ^18^F-FDG PET-CT group). Those patients who received no staging were treated with adjuvant systemic therapy in only 60.7% of the cases, compared to 73.4% in patients who were staged. There was no difference in the administration of adjuvant systemic therapy between the conventional imaging staged group and ^18^F-FDG PET-CT, 74.2% vs 71.4%, respectively (*P* = 0.588).

**Table 1:** Patient, tumor, and treatment characteristics categorized by preoperative staging procedure after diagnosis of ipsilateral breast tumor recurrence

Characteristics	IBTR		Conventional		^18^F-FDG PET-CT		None			
	*N* = 495	%	*N* = 229	%	*N* = 98	%	*N* = 168	%	Unadjusted *p* value	Adjusted *p* value
Primary surgery									**< 0.001**	**0.0167**
Mastectomy	59	11.9	35	15.3	19	19.4	5	3.0		
Breast-conserving surgery	436	88.1	194	84.7	79	80.6	163	97.0		
Primary ax. surgery									**0.0102**	0.8045
No axillary staging	30	6.1	7	3.1	4	4.1	19	11.3		
SLNB	194	39.2	90	39.3	43	43.9	61	36.3		
(c)ALND	271	54.7	132	57.6	51	52.0	88	52.4		
Primary nodal status									**< 0.001**	0.4613
Negative	357	72.1	172	75.1	65	66.3	120	71.4		
Positive	90	18.2	47	20.5	27	27.6	16	9.5		
Unknown	48	9.7	10	4.4	6	6.1	32	19.0		
Primary tumor size									**0.0026**	0.5508
< 20 mm	274	55.4	135	59.0	57	58.2	82	48.8		
21–50 mm	87	17.6	46	20.1	19	19.4	22	13.1		
Unknown	134	27.1	48	21.0	22	22.4	64	38.1		
Primary tumor grade									**< 0.001**	0.7498
I	77	15.6	40	17.5	18	18.4	19	11.3		
II	114	23.0	62	27.1	22	22.4	30	17.9		
III	68	13.7	35	15.3	21	21.4	12	7.1		
Unknown	236	47.7	92	40.2	37	37.8	107	63.7		
Hormone status primary									**0.0056**	0.7568
ER and PR negative	49	9.9	26	11.4	10	10.2	13	7.7		
ER/PR positive	252	50.9	130	56.8	52	53.1	70	41.7		
Unknown	194	39.2	73	31.9	36	36.7	85	50.6		
Time from primary surgery to IBTR diagnose										
Median, years	10.6 (0.4–31.8)		9.1 (0.5–30.0)		9.6 (0.4–30.2)		11.9 (0.4–31.8)		0.0913	0.765/0.415
Year of diagnosis									**< 0.001**	**0.5461**
0–124 pt. (2002–2010)	124	25.1	72	31.4	8	8.2	44	26.2		
124–248 pt. (2010–2011)	124	25.1	61	26.6	29	29.6	34	20.2		
248–372 pt. (2011–2013)	124	25.1	57	24.9	30	30.6	37	22.0		
372–496 pt. (2013–2014)	123	24.8	39	17.0	31	31.6	53	31.5		
Age IBTR, median years (range)										
Median, years	64.0 (26.0–93.0)		63.0 (27.0–93.0)		62.5 (26.0–81.0)		66.0 (37.0–88.0)		**0.004**	0.627/0.702
Receptor status IBTR									**0.019**	0.7829
Triple negative	62	12.5	32	14.0	13	13.3	17	10.1		
HRneg_Her2pos	17	3.4	10	4.4	4	4.1	3	1.8		
Hrpos_Her2pos	32	6.5	15	6.6	4	4.1	13	7.7		
HRpos_Her2neg	336	67.9	157	68.6	72	73.5	107	63.7		
Unknown	48	9.7	15	6.6	5	5.1	28	16.7		

*IBTR* ipsilateral breast tumor recurrence, ^*18*^*F-FDG PET*-*CT* 18-Fluorine 2-Fluoro-2-deoxy-D-Glucose positron emission tomography-computer tomography, *ax* axillary, (*c)ALND* (completion) axillary lymph node dissection, *SLNB* sentinel lymph node biopsy, *mm* millimeter, *ER* estrogen receptor, *PR* progesterone receptor, *HR* hormone receptor, *HER2* human epidermal growth receptor 2, *neg* negative, *pos* positive, *pt* patient

### Preoperative staging modalities

**Fig. 1:**
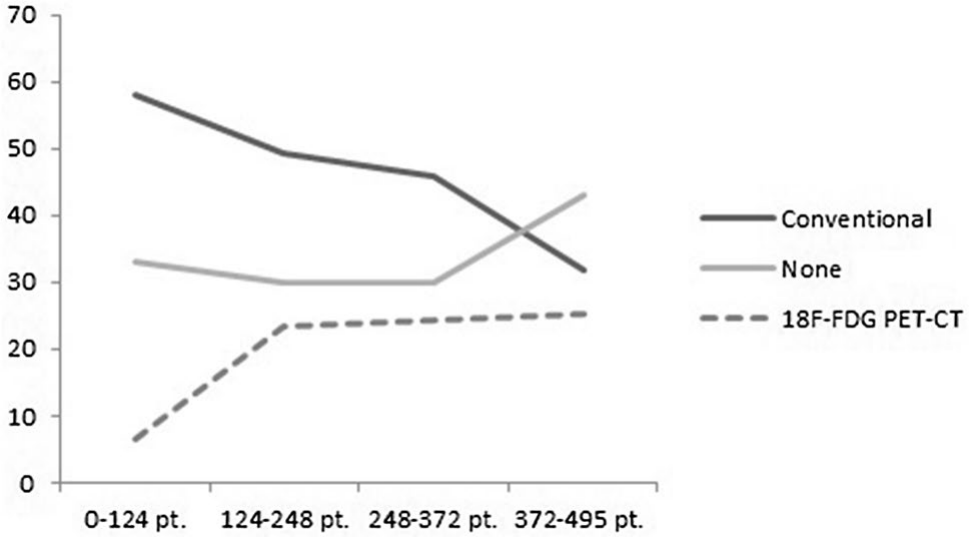
Percentages of IBTR patients staged with ^18^F-FDG PET-CT imaging versus conventional imaging versus no imaging over time. ^*18*^*F*-*FDG PET*-*CT* 18-Flurorine-2-Fluoro-2-deoxy-D-Glucose positron emission tomography-computer tomography. The group of 0−124 were the first 124 patient treated for IBTR in 2002−2010, second group of 124 patients: 124−248 treated in 2010−2011, third group: 248−372 treated in 2011−2013, and 372−495 the last group of patients treated in 2013−2014

Of the 495 patients, 229 patients (46.3%) underwent preoperative staging with conventional imaging, 89 patients (19.8%) with ^18^F-FDG PET-CT, and 168 (33.9%) received no preoperative staging imaging (*N* = 168) (Table 1). As shown in Fig. 1, the use of the different imaging procedures changed over time; the use of ^18^F-FDG PET-CT increased from 6.5% in 2008–2010 to 25.2% in 2013–2014, while the use of conventional imaging decreased from 58.1 to 31.7%. In 2008–2010, 33.1% of patients received no preoperative staging imaging, and this percentage fluctuated to 29.8% in 2010–2011, 29.8% in 2011–2013, and 43.1% in 2013–2014.

### Regional recurrences

Regional recurrences as first event after IBTR occurred in 16 patients after a median time of 2.2 years (range 0.4–7.0). In eight patients, the regional recurrence was located in the contralateral axilla (*N* = 8). The other recurrences were located in the ipsilateral supraclavicular nodal area (*N* = 2), ipsilateral axillary (*N* = 1), parasternal (*N* = 2) or contralateral infraclavicular nodal area (*N* = 1), or in multiple regional nodal areas (*N* = 2). The 6-year regional recurrence-free interval was 96.1% (CI 94.1%–98.1%). Patients screened with conventional imaging had a 6-year RR of 96.4% vs. 96.2% and 95.3% for patients with no imaging and ^18^F-FDG PET-CT (*p* = 0.766).

### Distant recurrences

After a median follow-up period of 4.9 years (range 0.3–13.2) after IBTR, 82 (16.5%) patients had experienced a distant recurrence, and in 10 (12.2%) of these patients a distant recurrence was diagnosed after a local re-recurrence (*N* = 4) or after a regional re-recurrence (*N* = 6). Predominant metastatic sites were the bones (30.6%; *N* = 22), the lungs (18.1%; *N* = 13), the liver (12.5%; *N* = 9), the brain (8.3%; *N* = 6), or to other places (12.5%; *N* = 9). In 13 patients, distant recurrences were found in multiple organs (18.1%; *N* = 13). Distant recurrence as first event occurred after a median time of 2.7 years (range 0.04–8.7) following treatment of IBTR. The mean time of developing a distant recurrence after IBTR did not significantly differ between patients screened with ^18^F-FDG PET-CT, conventional imaging, or no imaging (*p* = 0.648).

### Propensity score weighting, check PS weights, overlapping, and balance

The GBM was used and the convergence was achieved in all comparisons (Supplemental material, Fig. 3a, b, c). The overlap between the groups is shown in Table 1, but also in the box plots shown in Figs. 4a, b, c (Supplemental material) and was good to moderate. Next to the overlap, the balance was also checked and is shown in Fig. 5 (Supplemental material). The balance looks sufficient; however, the covariate primary surgery (breast-conserving therapy versus mastectomy) was still not balanced correctly after propensity score weighting.

### Baseline characteristics after PS weights

In Table 1, the adjusted *p* values, after adjusting the PS weights to the sample, are shown. After adjusting the PS weights, all the covariates, except for the covariate primary surgery, are no longer statistically significant and therefore balanced between the staging groups. Only the variable ‘primary surgery’ is taken as a covariate next to the PS weights in the cox regression model.

### *Survival curves of DRFI*

**Fig. 2:**
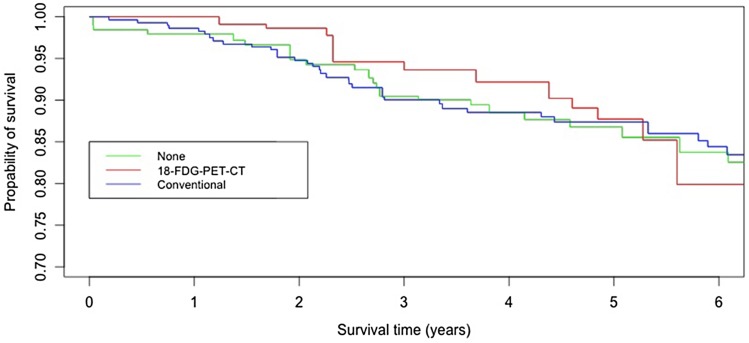
Propensity score weighted Kaplan–Meier curves of distant recurrence-free interval according to staging method groups. ^*18*^*F*-*FDG PET*-*CT* 18-Flurorine-2-Fluoro-2-deoxy-D-Glucose positron emission tomography-computer tomography

Figure 2 presents the Kaplan Meier curves for the three different staging modalities (without correcting for the unbalanced covariate ‘primary surgery’) and showed no impact of the different imaging groups on the DRFI. Finally, Cox regression analyses were performed with and without the unbalanced covariate ‘primary surgery.’ The different staging modalities had no significant effect on the DRFI with a HR 0.86 (95% CI 0.37–1.98) for ^18^F-FDG PET-CT compared to no imaging and HR 0.96 (95% CI 0.55–1.67) for conventional imaging compared to no imaging (Table 2). By adding the covariate primary surgery to the Cox regression model (because of the imbalance after weighting), the effect of the different imaging groups remained (Table 2); however, the proportional hazard (PH) assumption became questionable. To assure the PH assumptions that were made, sensitivity analyses were performed for the primary breast-conserving surgery group and showed no significant effect of the different staging modalities on the DRFI.

**Table 2:** Cox regression analyses for distant recurrence-free interval

	Coefficients	Standard error	*p* value	Hazard ratio (95% confidence interval)
Without covariate				
None	*Ref*			
^18^F-FDG PET-CT	− 0.15425	0.42637	0.718	0.8571 (0.3716–1.977)
Conventional	− 0.04582	0.28627	0.873	0.9552 (0.5450–1.674)
With covariate				
None	*Ref*			
^18^F-FDG PET-CT	− 0.2523	0.4568	0.581	0.7770 (0.3147–1.902)
Conventional	− 0.1398	0.3122	0.654	0.8695 (0.4716–1.603)
Primary surgery	− 0.6393	0.4219	0.130	0.5277 (0.2308–1.206)

^*18*^*F-FDG PET*-*CT* 18-Flurorine-2-Fluoro-2-deoxy-d-Glucose positron emission tomography-computer tomography

## Discussion

In this nationwide cohort of 495 patients included in the SNARB study with an ipsilateral breast tumor recurrence, 46.3% were preoperatively staged with conventional imaging, 19.8% with ^18^F-FDG PET-CT, and 33.9% received no preoperative imaging at all to detect distant metastases. Distant recurrences, as a first event after treatment of IBTR, occurred in 72 patients (14.5%). Propensity score analyses showed no difference in the likelihood of developing distant recurrence, according to the imaging strategy used at time of IBTR.

At baseline, all the covariates differed significantly between the three staging groups, except for the median time from primary surgery to IBTR diagnosis. To correct for these baseline differences, a PS analyses was used, with the aim to reduce or even eliminate confounding. PS methods are particularly appealing when multiple covariables are studied, and the number of events is rare [24, 25]. After propensity score analyses, we did not find a statistically significant difference in the risk of distant metastases between the three staging groups. This finding is in accordance with the results of a study by Neuman et al., who reported no difference in the risk of developing distant recurrence between patients with imaging versus without imaging at the time of locoregional recurrence [26].

Historically, IBTR has been considered a risk factor for distant recurrence and thus resulting in a poorer prognosis [26–28]. Synchronous distant metastases are reported in up to 15–30% of patients with an IBTR and up to 35% of patients with isolated lymph node recurrence [26]. Factors found to be associated with the presence of synchronous metastases are the TNM stage of the primary tumor, with patients with more advanced stages having a higher risk [29], and the type of locoregional recurrence, with patients with lymph node recurrences having a higher risk compared to patients with IBTR [26]. Because of this high risk of synchronous distant metastases, the recommendation is to stage all patients diagnosed with an IBTR for distant metastases.

In the current study, we did not observe a difference in the meantime to detection of distant recurrence, nor in the risk of regional recurrences after IBTR, between the different imaging strategies. Although current guidelines recommend the use of chest X-ray, liver ultrasonography or CT, and bone scintigraphy, these imaging modalities have been shown to be less sensitive and specific than ^18^F-FDG PET-CT [13]. Theoretically, occult metastases could be present at time of diagnosis of IBTR, though too small for detection on conventional imaging modalities. Staging using a more sensitive staging strategy, i.e., ^18^F-FDG PET-CT could prevent false-negative outcomes, by detecting the smaller distant metastasis. Synchronous small metastases missed at time of IBT could continue to grow, and could become clinical overt during follow-up. Thereby, ^18^F-FDG PET-CT is deemed to be especially valuable in the detection of extra-axillary nodal metastases [13, 30]. In the current study, the lack of difference in time to detection of distant recurrence and in the regional recurrence risk does not support these hypotheses.

Imperative for patients is the impact of distant staging on patients’ treatment plan. Changes in treatment plans could include initiation or avoidance of medical treatment such as hormone therapy and chemotherapy, but also surgical treatment of IBTR [7]. If extensive metastatic disease is diagnosed, patients are generally considered not curable and treatment is aimed to alleviate symptoms and, if possible, prolong survival [5, 7]. Omitting local treatment spares patients the morbidity associated with surgery and/or radiotherapy and minimizes the impact of these treatments on quality of life. Therefore, no staging at time of IBTR may lead to unnecessary exposure to potentially harmful local surgical procedures [31]. Furthermore, guidelines recommend treating patients with IBTR, especially those with estrogen receptor-negative tumors, with adjuvant chemotherapy, while in the case of synchronous metastasis the standard treatment with adjuvant chemotherapy will be postponed until having symptomatic metastasis. Neuman et al. found that 27% of 445 patients with a locoregional recurrence had synchronous distant metastases at time of their IBTR [26]. Hypothetically, choosing not to stage patients with IBTR will lead to overtreatment of up to those 27% of patients. The impact on patient management and patient’s physical as well as mental status underscores the need for accurate staging modalities in this group of IBTR patients who have a variety of treatment options available to them.

Over time, many studies presented the pros and cons of both ^18^F-FDG PET-CT and conventional imaging. ^18^F-FDG PET-CT is able to perform a whole-body evaluation in one session [5]. This will shorten the time until start of treatment and perhaps will reduce health care-related costs, due a to lower number of hospitals visits, additional diagnostic procedures, and unnecessary curative treatments [13]. However, further studies are needed to investigate the cost-effectiveness of ^18^F-FDG PET-CT, whether these possible lower costs would indeed outweigh the high costs of ^18^F-FDG PET-CT. As was shown in this study, the percentage of patients with isolated IBTR screened with ^18^F-FDG PET-CT increased over the years, while the use of conventional imaging decreased. Whether ^18^F-FDG PET-CT should replace conventional imaging in the preoperative staging of patients with IBTR should preferably depend on more aspects besides its diagnostic accuracy and cost-effectiveness, but also on the impact on prognosis and quality of life.

Some caveats apply to this study; because of its retrospective nature, reasons to use conventional imaging, ^18^F-FDG PET-CT, or no imaging for staging were not always reported in the patient files. All patients with IBTR and synchronous metastatic disease were not considered for inclusion and therefore not registered in the database. Data are lacking regarding the numbers of excluded patients and how these synchronous metastasis were detected. Further research is encouraged to evaluate the impact of the different imaging modalities on the prognosis and to determine factors associated with an increased risk of synchronous metastases. Lastly, the diagnostic accuracy of ^18^F-FDG PET-CT and conventional imaging modalities are likely to vary depending on the different techniques used between the different hospitals, regarding length of radioisotope uptake, image acquisition time, and the mode of image interpretation. Nonetheless, the current study is based on the largest cohort of patients with an IBTR, as far as we are aware of. Furthermore, this is a multicenter, nationwide study providing data of different types of hospitals in the Netherlands, representative for IBTR patients in daily practice.

## Conclusions

This study showed a wide variation in the use of imaging modalities for staging of IBTR patients in the Netherlands. After propensity score weighting, no statistically significant impact of the different imaging modalities on the DRFI was shown. Based on these results, it is not possible to recommend staging for distant metastases using ^18^F-FDG PET-CT over conventional imaging techniques.

## Electronic supplementary material

Below is the link to the electronic supplementary material.

Fig. 3a-b-c Balance assessment. Each panel presents balance measures for one of the staging methods. The top panel presents no staging, the middle panel presents 18F-FDG PET-CT and the bottom panel presents conventional staging Supplementary material 1 (JPG 43 KB)

Supplementary material 2 (JPG 44 KB)

Supplementary material 3 (JPG 45 KB)

Fig. 4a-b-c Overlap assessment. Each panel presents box plots of the estimated propensity scores for oneof the staging methods. The top panel presents no staging, the middle panel presents 18F-FDG PET-CTand the bottom panel presents conventional staging Supplementary material 4 (JPG 45 KB)

Supplementary material 5 (JPG 46 KB)

Supplementary material 6 (JPG 36 KB)

Fig. 5 Effect size plots for assessing the balance of covariables on patients. The lines in the balance plots connect the values for the same variable before and after weighting. A closed redcircle indicates a covariate for which the difference between the group means is statistically significant, and anopen red circle means no statistically significant difference. A red line identifies a variable for which thestandardized bias or effect size increases with weighting; a blue line identifies a variable for which balanceimproves with weighting.Supplementary material 7 (JPG 21 KB)
