# Supplementary figures and images for: Screening for distant metastases in patients with ipsilateral breast tumor recurrence: the impact of different imaging modalities on distant recurrence-free interval
Source: Breast Cancer Res Treat. 2019 Apr 6;175(2):419–28. doi: 10.1007/s10549-019-05205-z (PMC6533220; doi:10.1007/s10549-019-05205-z)

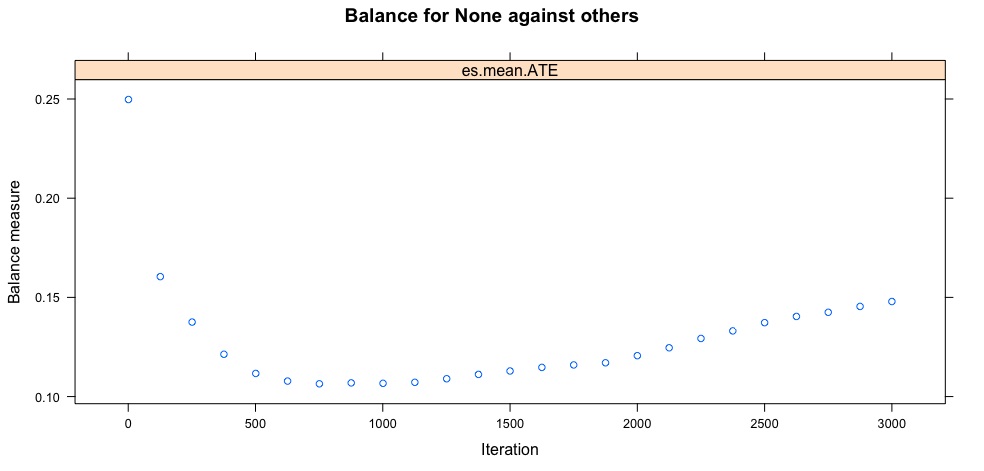

Supplement: Supplementary file 1 — Fig. 3a-b-c Balance assessment. Each panel presents balance measures for one of the staging methods. The top panel presents no staging, the middle panel presents 18F-FDG PET-CT and the bottom panel presents conventional staging Supplementary material 1 (JPG 43 KB) [file 10549_2019_5205_MOESM1_ESM.jpg]

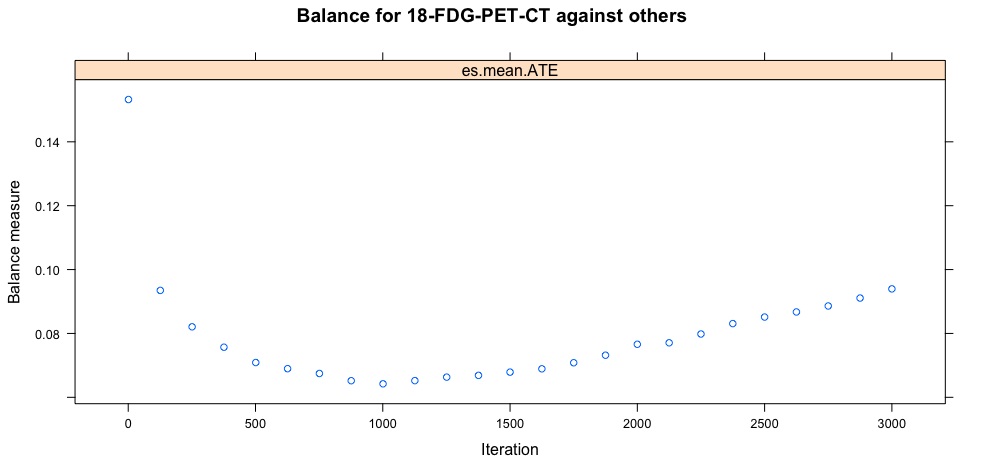

Supplement: Supplementary file 2 — Supplementary material 2 (JPG 44 KB) [file 10549_2019_5205_MOESM2_ESM.jpg]

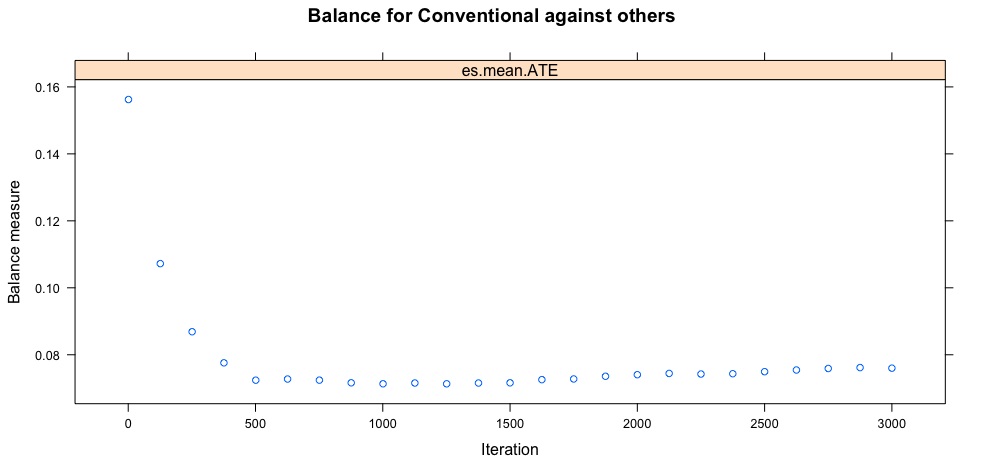

Supplement: Supplementary file 3 — Supplementary material 3 (JPG 45 KB) [file 10549_2019_5205_MOESM3_ESM.jpg]

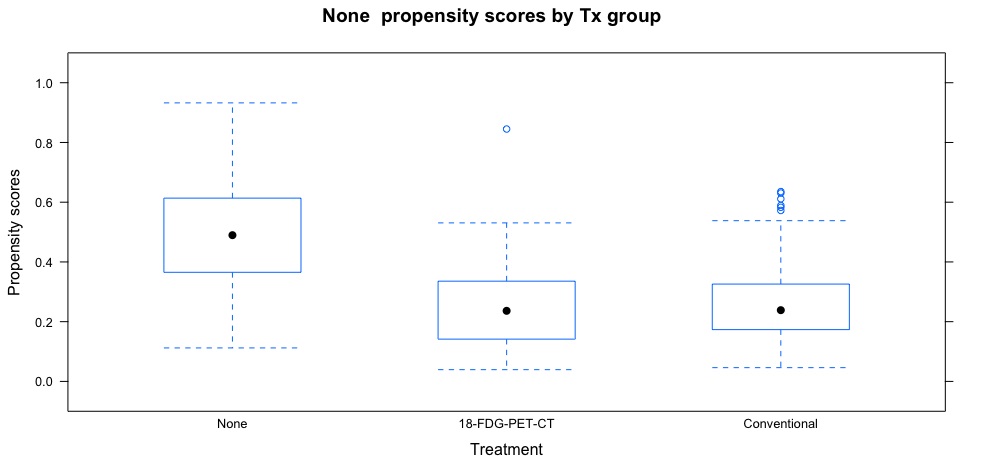

Supplement: Supplementary file 4 — Fig. 4a-b-c Overlap assessment. Each panel presents box plots of the estimated propensity scores for oneof the staging methods. The top panel presents no staging, the middle panel presents 18F-FDG PET-CTand the bottom panel presents conventional staging Supplementary material 4 (JPG 45 KB) [file 10549_2019_5205_MOESM4_ESM.jpg]

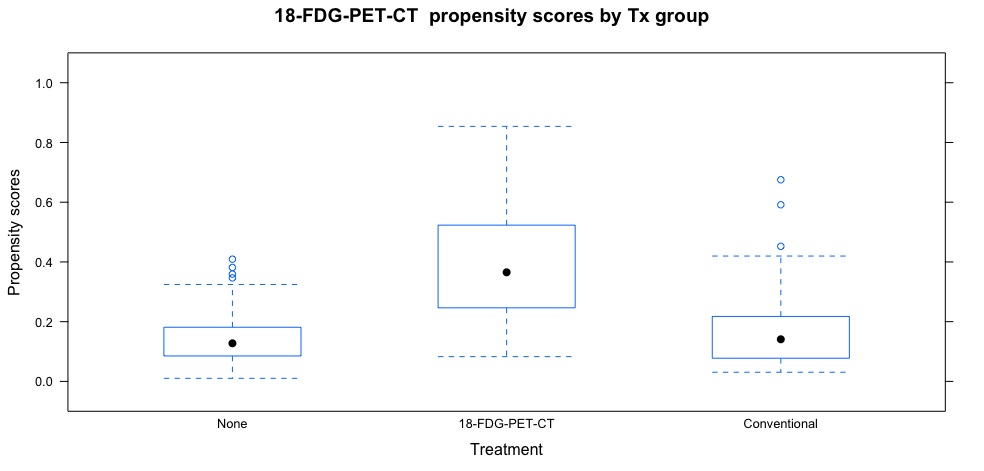

Supplement: Supplementary file 5 — Supplementary material 5 (JPG 46 KB) [file 10549_2019_5205_MOESM5_ESM.jpg]

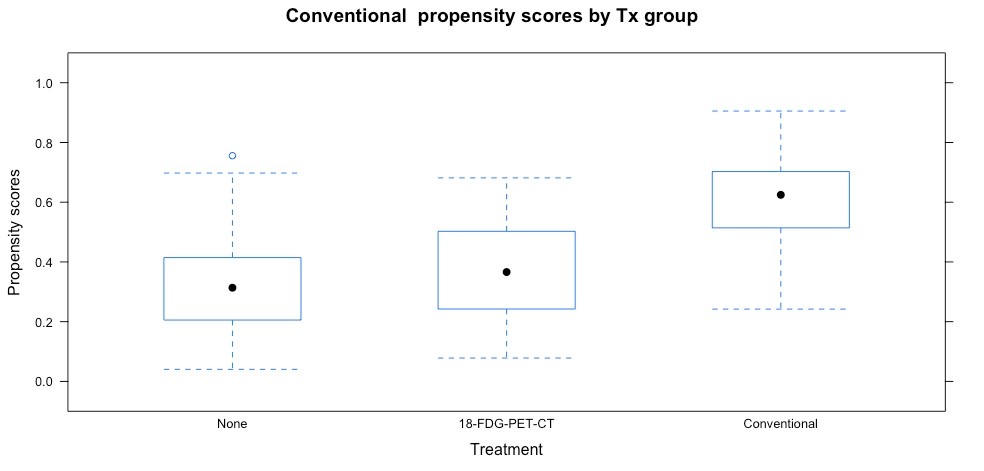

Supplement: Supplementary file 6 — Supplementary material 6 (JPG 36 KB) [file 10549_2019_5205_MOESM6_ESM.jpg]

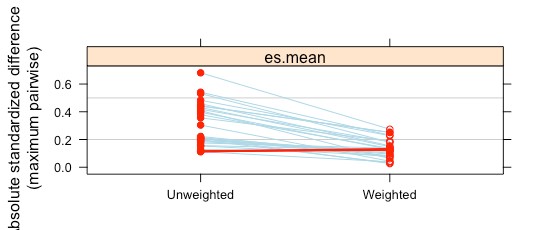

Supplement: Supplementary file 7 — Fig. 5 Effect size plots for assessing the balance of covariables on patients. The lines in the balance plots connect the values for the same variable before and after weighting. A closed redcircle indicates a covariate for which the difference between the group means is statistically significant, and anopen red circle means no statistically significant difference. A red line identifies a variable for which thestandardized bias or effect size increases with weighting; a blue line identifies a variable for which balanceimproves with weighting.Supplementary material 7 (JPG 21 KB) [file 10549_2019_5205_MOESM7_ESM.jpg]
